# Investigating *APOE*, APP-Aβ metabolism genes and Alzheimer’s disease GWAS hits in brain small vessel ischemic disease

**DOI:** 10.1038/s41598-020-63183-5

**Published:** 2020-04-28

**Authors:** Sonja Blumenau, Marco Foddis, Susanne Müller, Manuel Holtgrewe, Kajetan Bentele, Daniel Berchtold, Dieter Beule, Ulrich Dirnagl, Celeste Sassi

**Affiliations:** 1 Department of Experimental Neurology, Center for Stroke Research Berlin (CSB), Charité - Universitätsmedizin Berlin, Corporate Member of Freie Universität Berlin, Humboldt-Universität zu Berlin, and Berlin Institute of Health, Berlin, Germany; 2 Berlin Institute of Health, BIH, Core Unit Bioinformatics, Berlin, Germany

**Keywords:** Gene expression, Genetic association study, Genotype, Genetics, Neuroscience, Neurology

## Abstract

Alzheimer’s disease and small vessel ischemic disease frequently co-exist in the aging brain. However, pathogenic links between these 2 disorders are yet to be identified. Therefore we used Taqman genotyping, exome and RNA sequencing to investigate Alzheimer’s disease known pathogenic variants and pathways: *APOE* ε4 allele, APP-Aβ metabolism and late-onset Alzheimer’s disease main genome-wide association loci (*APOE*, *BIN1*, *CD33*, *MS4A6A*, *CD2AP*, *PICALM*, *CLU*, *CR1*, *EPHA1*, *ABCA7)* in 96 early-onset small vessel ischemic disease Caucasian patients and 368 elderly neuropathologically proven controls (HEX database) and in a mouse model of cerebral hypoperfusion. Only a minority of patients (29%) carried *APOE* ε4 allele. We did not detect any pathogenic mutation in *APP*, *PSEN1* and *PSEN2* and report a burden of truncating mutations in APP-Aß degradation genes. The single-variant association test identified 3 common variants with a likely protective effect on small vessel ischemic disease (0.54>OR > 0.32, adj. p-value <0.05) (*EPHA1* p.M900V and p.V160A and *CD33* p.A14V). Moreover, 5/17 APP-Aß catabolism genes were significantly upregulated (LogFC > 1, adj. p-val<0.05) together with *Apoe*, *Ms4a* cluster and *Cd33* during brain hypoperfusion and their overexpression correlated with the ischemic lesion size. Finally, the detection of Aβ oligomers in the hypoperfused hippocampus supported the link between brain ischemia and Alzheimer’s disease pathology.

## Introduction

Late-onset sporadic Alzheimer’s disease (LOAD) and small vessel ischemic disease (SVID) frequently influence each other and co-exist in the aging brain depicting a clinical, neuroradiological and neuropathological spectrum defined as ‘mixed dementia’. Although mixed dementia represents the second common form of dementia in the elderly, as over 45% of LOAD patients neuropathologically diagnosed displayed significant cerebrovascular pathology^1^, the nature and the pathogenic ground at the basis of AD-SVID interaction is poorly understood^2^. *APOE* ε4 allele is the strongest risk factor for sporadic LOAD^3–5^, however its role in SVID has not been extensively investigated. Common hallmark in small vessel disease is cerebral amyloid angiopathy (CAA), which is caused by excessive deposition of Aβ 40 and 42 on the walls of small vessels^6,7^, responsible both for its ischemic and hemorragic manifestations (SVID and intracerebral hemorrhage [ICH])^8^. Both rare familial and common sporadic small vessel disease cases pointed to the potential role of APP-Aß dysmetabolism as key pathogenic mechanism underlying CAA small vessel disease subtype. First, autosomal dominant fully penetrant mutations in the secretase domain of APP, *APP* duplication, *CST3* and *TTR* rare mutations cause familial CAA^9–11^. Second, common variants in *IDE* and *LRP1* have been associated with increased risk of diabetes type 2 and migraine, respectively, that frequently are co-morbidities in SVID patients^12,13^. Third, perivascular and parenchymal Aß deposits have been reported in genetically diagnosed CADASIL patients and vascular dementia cases^14–17^. Despite the growing body of evidence supporting an imbalance between Aß production and degradation, APP-Aß metabolism role in SVID remains unknown.

Finally, in the last 10 years 9 main LOAD genome-wide association study (GWAS) loci have been identified and replicated by at least 2 independent GWASs and present the strongest effect sizes after *APOE* (*BIN1*, *CLU*, *CR1*, *PICALM*, *MS4A6A*, *ABCA7*, *EPHA1*, *CD33*, *CD2AP*). They shed light on critical LOAD pathogenic pathways and these include: immune response (*MSA4A* cluster, *CD33*, *CR1*, *EPHA1, CD2AP, ABCA7*), Aβ40–42 clearance (*PICALM*, *BIN1, CD33 and ABCA7)*, lipid metabolism (*CLU*, *ABCA7*) and vesicles trafficking (*PICALM*, *BIN1*) (http://www.alzgene.org/). Among the genetic mechanisms underlying an increased susceptibility for LOAD at these loci, coding variability is emerging as a critical factor^18–21^.

**Figure 1:**
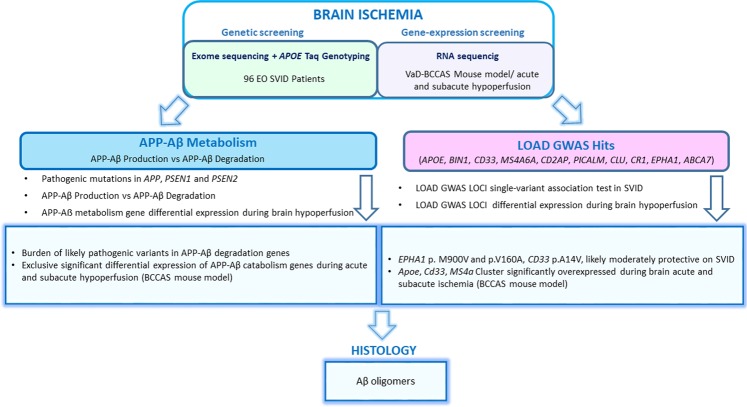
Pipeline followed in the study. SVID, small vessel ischemic disease; VaD, vascular dementia; BCCAS, bilateral common carotid artery stenosis; LOAD, late-onset Alzheimer’s disease; GWAS, genome-wide association study.

Therefore, in this study we investigated *APOE ε2, ε3* and *ε4* alleles, APP-Aβ metabolism genes and the most replicated AD GWAS loci through a genetic screening in 96 early-onset independent familial and apparently sporadic SVID Caucasian patients and 368 elderly neuropathological proven controls (HEX database) and through a differential gene expression study during acute and subacute brain ischemia in a mouse model of vascular dementia and subcortical ischemic stroke. Moreover, we analysed whether brain hypoperfusion may have contributed to the generation of AD neuropathological hallmarks (Fig. 1).

We hypothesize that 1) coding variability together with significant differential gene expression in APP-Aß metabolism genes and LOAD GWAS loci may play a role in SVID and brain ischemia and 2) acute severe hypoperfusion-ischemia may prime APP misfolding, toxic soluble oligomers formation that may in the long term accumulate in the stable form of amyloid plaques, as described in elderly patients with vascular dementia^22,23^.

## Materials and Methods

### Gene selection

We studied *APOE* ε2, ε3 and ε4 genotype and 2 clusters of genes: 1) APP-Aβ metabolism genes: 31 genes involved in Aβ production (*APP*, *PSEN1*, *PSEN2*, *ADAM9*, *ADAM10*, *ADAM17*, *BACE1*, *BACE2*, *NCSTN*, *PSENEN*, *APH1B, MEP1B, GPR3*), APP stabilization (*APLP1*, *APBA1)*, APP recycling (*SORL1*), Aβ deposition (*TTR*), intracellular degradation (*ECE1*, *ECE2*, *IDE*, *CST3*, *CTSB*, *CTSD*, *LYZ*, *MME)*, extracellular degradation and clearance (*ACE*, *MMP3*, *A2M*, *PLAT*, *KLK6*, *LRP1)* and 2) LOAD GWAS mainly replicated loci: *APOE*, *BIN1*, *CLU*, *CR1*, *PICALM*, *MS4A6A*, *ABCA7*, *EPHA1*, *CD33*, *CD2AP*. Selection criteria for these genes has already been reported^19,24^. The pipeline followed in this study is described in Fig. 1.

### Patient cohort

The cohort was composed of 96 independent familial and early-onset apparently sporadic SVID Caucasian non-Hispanic cases from the US, NINDS (National Institute of Neurological Disorders and Stroke), whose DNA was extracted and collected at the NINDS Repository.

All NINDS Repository samples were collected only after an IRB-approved, signed informed consent was secured by the submitter. All methods were carried out in accordance with relevant guidelines and regulations.

Inclusion criteria included small vessel ischemic disease diagnosis based on TOAST classification, early age at onset (<65 years [only 2 cases, whose age-at onset was 68 and 71 years old have been included in the study because familial]), absence of known pathogenic mutations in Mendelian small vessel disease genes (*HTRA1*, *NOTCH3*, *ACTA2* and *COL4A1*) and no enrichment for vascular risk factors except for hypertension, which generally plays a critical role in elderly people^12^. The mean age at disease onset was 51.5 years (range 34–71 years). 82.3% of the cases were male and 44.8% of the cases were positive for a familial history of cerebrovascular disorders. Among the comorbidities and possible risk factors for SVID, hypertension was reported in 60.4% of the patients, diabetes type 2 in 30.2%, myocardial infarction in 7.3%. The majority of the patients (at least 88.54%) did not present atrial fibrillation (AF), which is among the most important risk factors for embolic small vessel occlusion^25^. In 4.1% and 7.3% of the patients the presence of AF was reported and unknown, respectively. Given the prevalent role of hypertension and type 2 diabetes in SVID in the elderly people^12^ and the young age at onset of the cohort, these patients were considered enriched for genetic risk factors (Table 1). Finally, 368 controls>70 years of age were selected from ‘HEALTHY EXOMES’, HEX, a publicly available database, which collects exome sequencing data from elderly neuropathologically proven controls (https://www.alzforum.org/exomes/hex)^26^.

**Table 1:** Discovery cohort.

SVID	SEQUENCING STRATEGY	ORIGIN	AGE at onset (YRS)	MALE (%)	Familial(%)	*APOE* ε4* (%)	Hypertension	Diabetes	MI	AF +/-/NA	Hypercholesterolemia
			MEAN ± SD(RANGE)								
96	WES	Caucasian, non-Hispanic (US)	51.5 (34–71)	82.3	44.8	29	60.4	30.2	7.3	4	2

WES, whole exome sequencing; YRS, years; MI, myocardial infarction; AF, atrial fibrillation; *at least one *APOE* ε4 allele; NA, not available.

### Exome sequencing in patients

We performed whole exome sequencing on a cohort of 96 independent familial and early-onset sporadic SVID cases. DNA was extracted from blood using standard protocols. Library preparation for next generation sequencing used 50 ng DNA. Exome libraries were prepared using Nextera® Rapid Capture Exome Kit (4 rxn × 12 plex, FC-140-1002). The DNA library was then hybridized to an exome capture library (Nextera, Illumina Inc.) and precipitated using streptavidin-coated magnetic beads (Nextera, Illumina). Exome-enriched libraries were PCR-amplified, and then DNA hybridized to paired-end flow cells using a cBot (Illumina, Inc.) cluster generation system. Samples were sequenced on the Illumina HiSeq™ 3000/4000 using 2×76 paired end reads cycles.We used exome sequencing data to identify common (minor allele frequency [MAF] > 3%), rare (MAF < 3%), and very rare (MAF < 1%) coding variants in 31 genes involved in APP-Aβ metabolism (*A2M* [NM_000014], *ACE* [NM_000789], *ADAM9* [NM_003816], *ADAM10* [NM_001110], *ADAM17* [NM_003183], *APBA1* [NM_001163], *APH1B* [NM_031301], *APLP1* [NM_001024807], *BACE1* [NM_012104], *BACE2* [NM_012105], *CST3* [NM_000099], *CTSB* [NM_001908], *CTSD* [NM_001909], *ECE1* [NM_001397], *ECE2* [NM_014693], *GPR3* [NM_005281], *IDE* [NM_004969], *LRP1* [NM_002332], *KLK6* [NM_001012964], *LYZ* [NM_000239], *MEP1B* [NM_005925], *MME* [NM_000902], *MMP3* [NM_002422], *NCSTN* [NM_015331], *PLAT* [NM_000930], *PSENEN* [NM_172341], *SORL1* [NM_003105], *TTR* [NM_000371], *APP* [NM_000484.2], *PSEN1* [NM_000021.2] and *PSEN2* [NM_000447.1]) and 9 main LOAD candidate genes (*ABCA7* [NM_019112]; *CD2AP* [NM_012120]; *MS4A6A* [NM_152851]; *CR1* [NM_000573]; *BIN1* [NM_139343]; *PICALM* [NM_001206946]; *EPHA1* [NM_005232]; *CLU* [NM_001831]; *CD33* [NM_001772]). The coding variants detected in these genes have been collected and analyzed. (Tables S1 and S2).

### Bioinformatics, exome sequencing

The reads were aligned using BWA-MEM v0.7.15^27^ to the reference GRCh37 (hs37d5.fa), separate read groups were assigned for all reads from one lane, and duplicates were masked using Samblaster v0.1.24^28^. Standard QC was performed using FastQC (http://www.bioinformatics.babraham.ac.uk/projects/fastqc). The variants were then called using GATK UnifiedGenotyper v3.7^29^ and annotated using Jannovar v0.24^30^ using RefSeq v105 exons.

We used Gene Ontology (http://www.geneontology.org/) and the human lysosome gene database (http://lysosome.unipg.it/) to select lysosomal genes in our dataset.

### *APOE* Genotyping

*APOE* genotypes comprising the *APOE* ɛ2, ɛ3 and ɛ4 alleles, were assayed using LightCycler 480 Instrument II (Roche). SNP-specific primers and probes were designed by Thermo Fisher (TaqMan genotyping assays). The polymorphisms distinguish the ɛ2 allele from the ɛ3 and ɛ4 alleles at amino acid position 158 (rs7412) and the ɛ4 allele from the ɛ2 and ɛ3 alleles at amino acid position 112 (rs429358).

### Statistical analysis

Power calculation was performed for Fisher´s exact test based on allelic association. We had 80% power for the detection of common variants (minor allele frequency [MAF] > 3%) with strong effect (OR < 0.6 or>2), with a significance value of two-sided α = 0.05 (Fig. S1).

In the single-variant analysis, allele frequencies were calculated for each coding variant in cases and controls and Fisher’s exact test on allelic association was performed. Fisher’s exact test was also used for the statistical analysis of the truncating mutations in APP-Aß metabolism genes. Low frequency and rare variants were defined as having a 1%<MAF < 3% and MAF < 1%, respectively, either in cases or controls. MAF was based either on HEX database for elderly controls>70 years of age or EXac database version 0.3.1 database (http://exac.broadinstitute.org/). A p-value of 0.05 was set as a nominal significance threshold, after false discovery rate (FDR) correction. We report the complete list of coding variants detected in the APP-Aβ metabolism genes and LOAD GWAS loci in the supplementary tables (Table S1 and S2).

T-test performed was used to detect the statistical significance of the number of neurons and glial cells positive for Aß oligomers in hippocampus during acute (2d) and subacute (7d) hypoperfusion in BCCAS and naive mice.

R version 3.3.2 (2016-10-31) (https://www.r-project.org/) was used for computations and graphs, particularly statmod-package v1.4.32 (power calculation) and Ggplot2 (graphs).

### BCCAS mouse model, experimental design and exclusion criteria

All experiments and experimental protocols were approved by the Landesamt für Gesundheit und Soziales and conducted according to the German Animal Welfare Act and institutional guidelines. 22 male C57BL/6 J mice, purchased at 8 weeks of age, Charles River, Germany, were housed in a temperature (22 ± 2 °C), humidity (55 ± 10%), and light (12/12-hour light/dark cycle) controlled environment. As previously described^31^, the animals underwent hypoperfusion between 9 and 13 weeks of age. Hypoperfusion was achieved by bilateral common carotid artery stenosis (BCCAS).

BCCAS mice were imaged before surgery, 24 hours and 1 week post-surgery. At 2 days and 7 days tissue was processed for immunohistochemistry and RNA sequencing.

The BCCAS surgery is further described in the supplementary.

### RNA sequencing data: acute (2d) and subacute (7d) hypoperfusion in BCCAS mouse model

To study APP-Aβ metabolism and LOAD GWAS genes during brain acute and subacute hypoperfusion, we used a mouse model of vascular dementia, where brain hypoperfusion is achieved through the placement of microcoils around both common carotid arteries leading to a ≈ 70% stenosis (bilateral common carotid stenosis [BCCAS] mouse model)^32^. The main features of the model during severe acute and subacute hypoperfusion have been already described^31^.

In this study, 8 BCCAS mice, 8 sham and 4 naive mice were sacrificed with cervical dislocation 2 days and 7 days post coil insertion surgery, followed immediately by post-mortem dissection of the prefrontal cortex, striatum and hippocampus from one hemisphere. The other hemisphere was preserved for immunohistochemistry. The dissected tissues were immersed in RNA later and stored at −80 °C for later use for mRNA-Sequencing. Total RNA was extracted using miRNeasy Kit (Qiagen, Cat # 217004). Total RNA quality was assessed with the use of Bioanalyzer. Average RIN (RNA Integrity Number) of our samples was 9. Next Generation Sequencing mRNA libraries were prepared with Illumina TruSeq RNA Library Preparation Kit (Illumina, Cat # RS-122-2001).

### Bioinformatics, RNA sequencing

Processing, quality assessment and analysis of RNAseq data was carried out using a custom pipeline. We aligned paired end reads with STAR^33^ against the GRCm38.p4 genome using gencode.vM12 annotation^34^ (http://www.gencodegenes.org/mouse_releases/12.html), excluding alternative scaffolds and patches. Gene counts were determined using HTSeq.^35^. Testing for differential gene expression and cerebral blood flow and gene-expression correlation was done using DESeq. 2^36^. Genes were counted as differentially expressed where they had a moderated fold change of 2 or more, contrasting coil to shame samples and where their false discovery rate (FDR) adjusted p-value was below 0.05.

### BCCAS mouse model, histology

The staining protocol for the mouse brain histological sections has been already described^31^. Briefly, for the 20 mice subjected to gene-expression study (8 BCCAS mice, 8 Sham mice and 4 naive mice) one hemisphere was used for RNA sequencing and the contralateral for histology. Fresh frozen hemispheres were cut into 20-µm-thick sections on a cryostat. Moreover, to histologically study both hemispheres, 2 mice were deeply anaesthetized with ketamine and xylazine and perfused through the heart with physiological saline followed by 4% paraformaldehyde, Alexa Fluor® 680 conjugate of WGA (Termofisher, W32465), 3% Gelatin (Sigma-Aldrich, G1890), 1% low melting agarose (Sigma Aldrich A4018) and 0.1% Evans Blue (Sigma Aldrich E2129). Subsequently, the brains were post-fixed for 24 hours in 4% PFA, and cryoprotected in 30% sucrose solution. PFA perfused brains were cut into 50-µm-thick on a cryostat. After washing with phosphate-buffered saline (PBS), free-floating sections were incubated with 10% normal goat serum (NGS, GeneTech, GTX27481) and 0.1% Triton-X-100 (Sigma-Aldrich, X100) in PBS for 1 h at room temperature to block unspecific binding. Primary and secondary antibodies were diluted in 1% NGS and 0.1% Triton-X-100 in PBS. Sections were incubated with rabbit anti-Aß oligomers primary antibody (abcam, Ab126892) and rat anti-GFAP primary antibody (Millipore, 345860) for astrocytes at 4 °C overnight. After thorough washing, sections were incubated at room temperature with AlexaFluor-594- conjugated goat anti-rat (Invitrogen, catalog #A11081) and AlexaFluor- 488-conjugated goat anti-rabbit (Invitrogen, catalog #A11034) secondary antibodies for 2 h at room temperature. Nuclei were counterstained with DAPI (Fluka, 32670). Sections were mounted with anti-fading mounting medium Shandon Immuno Mount (Thermo Scientific, 9990402) on Super Frost Plus glass slides (R.Langenbrinck, 03-0060). Microphotographs were taken with a confocal microscope (Leica TCS SPE; RRID: SciRes_000154).

### Oligomers detection and counts

ImageJ version 1.52 A was used to count neuronal and glial cells positive for Aβ oligomers.

### Methods to prevent bias, statistics

Mice were randomized to receive hypoperfusion. RNA library preparation and pooling were randomized and blinded, respectively. All methods were carried out in accordance with relevant guidelines and regulations.

## Results

### Genetic screening

#### *APOE* ε4 allele is not associated to increased risk for SVID

The majority of SVID cases were homozygous for ε3 allele (58.3%), around one third of the patients carried in heterozygosity the ε4 allele (27% and 1%, genotype frequencies for ε4/ε3 and ε4/ε2, respectively), whereas a minority of cases (1%) were homozygous either for *APOE* ε2 or ε4 allele (Table 2). Average age at onset for carriers was 51 years, which did not differ significantly compared to patients homozygous for ε3 allele (52 years). Finally, familial cases displayed a moderately higher *APOE* ε4 carrier frequency compared to sporadic ones (32.5% and 26.4%, respectively). *APOE* ε2 alelle was detected in 13/96 (13.5%) patients, 10/13 (77%) with a very young age at onset (≤55 years).

**Table 2:** *APOE* genotype.

COHORTS	N	ε4/ε4	*APOE* GENOTYPE (%)				
			ε4/ε3	ε3/ε3	ε3/ε2	ε2/ ε4	ε2/ ε2
Caucasian SVID	96	1	27	58.3	11.45	1	1
Caucasian controls*	6262	1.8	21.3	60.9	12.7	2.6	0.8
Caucasian LOAD*	5107	14.8	41.1	36.4	4.8	2.6	0.2

N, number; SVID, small vessel ischemic disease; LOAD, late-onset Alzheimer's disease. *Data for Caucasian controls and LOAD are taken from a previous publication (Farrer *et al*., 1997)

#### *APP-Aβ metabolism genes*

To study a possible role of APP-Aβ metabolism genes in SVID, we focused on 1) possible enrichment for pathogenic mutations in Mendelian genes (*APP*, *PSEN1* and *PSEN2*), underpinning autosomal dominant AD (http://www.molgen.ua.ac.be/ADMutations/) and 2) burden of damaging mutations in APP-Aβ catabolism genes upon APP-Aβ production genes, underlying sporadic LOAD^37^.

We screened protein coding variability in 31 genes involved in APP-Aß metabolism and we identified 130 coding variants: 21 common and 88 rare. *ADAM10* and *PSENEN* did not harbour any coding variant. Among the rare variants we report 21 novel variants, and 10 truncating mutations. The majority of the variants detected were singletons (86/130 [66.15%]). *BACE1*, *BACE2*, *CST3*, *CTSB* and *CTSD* did not harbour any rare damaging variant. The majority of patients, 75/96 (78.12%), carried at least one rare likely damaging variant and almost half of them, 43/96 (44.8%), harboured multiple likely pathogenic alleles in the studied genes.

#### *ADMendelian genes:APP*, *PSEN1*, *PSEN2*

We report a total of 5 rare coding variants in *APP*, *PSEN1* and *PSEN2*. None of these are likely to be deleterious: *APP* p.V576I and p.T280del do not cluster in the conserved secretase domain; *PSEN1* p.E318G and *PSEN2* p.L2F and p.R62H map outside the alpha helix surface of the transmembrane domains (TMs), where all the pathogenic mutations have been reported (alpha-helix rule)^38^ (Table 3).

**Table 3:** Coding variants detected in *APP*, *PSEN1* and *PSEN2* in the SVID cohort.

Gene	Position	rsID	Ref/Alt	Genomic change	Aa change	PROVEAN	SIFT	Polyphen2	Carrier freq (%)	MAF(%)	Carrier *APOE* ε4	Hex70 (%)
*APP*	21: 27284236	rs200769792	C/T	c.1726G > A	p.V576I	Neutral	Tolerated	probably damaging	1/96 (1)	0.5	e3/e2	—
*APP*	21: 27394181	rs764406483	TGTG/T	c.837_839del	p.T280del	NA	NA	NA	2/96 (2)	1	e3/e3 e3/e3	—
*PSEN1*	14: 73673178	rs17125721	A/G	c.953 A > G	p.E318G	Deleterious	Damaging	benign	5/90 (5.5)	2.7	e4/e3 e3/e3 e3/e3 e4/e3 e3/e3	2
*PSEN2*	1: 227069612	NOVEL	C/T	c.4 C > T	p.L2F	Neutral	Damaging	probably damaging	1/93 (1)	0.5	e3/e3	—
*PSEN2*	1: 227071449	rs58973334	G/A	c.185 G > A	p.R62H	Neutral	Tolerated	benign	1/93 (1)	0.5	e3/e3	—

Aa, amino acid; freq, frequency,  NA, not available.

#### *Other genes playing a key role in APP-Aß metabolism. CST3 p.A25T in homozygosity and SORL1 variants clustering in VPS domain are known risk factors for LOAD and may influence SVID susceptibility*

Among the variants detected in the other genes involved in APP-Aß metabolism, 3 missense mutations in genes playing a role in APP-Aß degradation were of particular interest: one polymorphism reported as pathogenic in the ClinVar database (https://www.ncbi.nlm.nih.gov/clinvar/), *CST3* p.A25T, and *SORL1* p.E270K and p.A528T (Table 1).

Importantly, homozygosity for *CST3* p.A25T has been significantly associated with AD^39^ and other neurodegenerative conditions such as macular degeneration^40^. In our cohort, we report 4/96 [4.16%] patients homozygous for the minor allele A, a carrier frequency which was 2.55 times higher when compared with HEX controls (6/368 [1.63%]). Notably, the homozygous carriers, 3 sporadic and 1 familial cases, displayed an average age at onset of 49.75 years (range: 39–60 y), 3/4 (75%) were homozygous for *APOE* ε3 allele and presented a MMSE score moderately lower (26 < MMSE < 28). By contrast, only one case carried in heterozygosity the *APOE* ε4 allele. Finally, among *CST3* p.A25T homozygous carriers only 1/4 (25%) patients presented 3 moderate risk factors for SVID: hypertension, myocardial infarction and type 2 diabetes, 3/4 (75%) displayed hypertension and 1/4 (25%) did not present any risk factor for SVID (Table S3).

Interestingly, *SORL1* p.E270K and p.A528T clustering in the vacuolar protein sorting (VPS10) domain (Aa 124–757) have been found in 2/96 SVID patients homozygous for *APOE* ε3 allele and have been reported pathogenic and to segregate within AD families^41^.

#### *APP-Aβ genes, pooled variants. Analogously to LOAD, SVID patients are enriched for LoF mutations in genes involved in APP-Aß degradation rather than production*

We then compared coding genetic variability between 14 genes mainly involved in APP-Aβ production (*ADAM10*, *ADAM17*, *ADAM9*, *APBA1*, *APH1B*, *APLP1*, *APP*, *BACE1*, *BACE2*, *GPR3*, *NCSTN*, *PSEN1*, *PSEN2*, *PSENEN*) and 17 genes taking part in APP-Aß degradation (*A2M*, *ACE*, *CST3*, *CTSB*, *CTSD*, *ECE1*, *ECE2*, *IDE*, *KLK6*, *LRP1*, *LYZ*, *MEP1B*, *MME*, *MMP3*, *PLAT*, *SORL1*, *TTR*).

**Figure 2:**
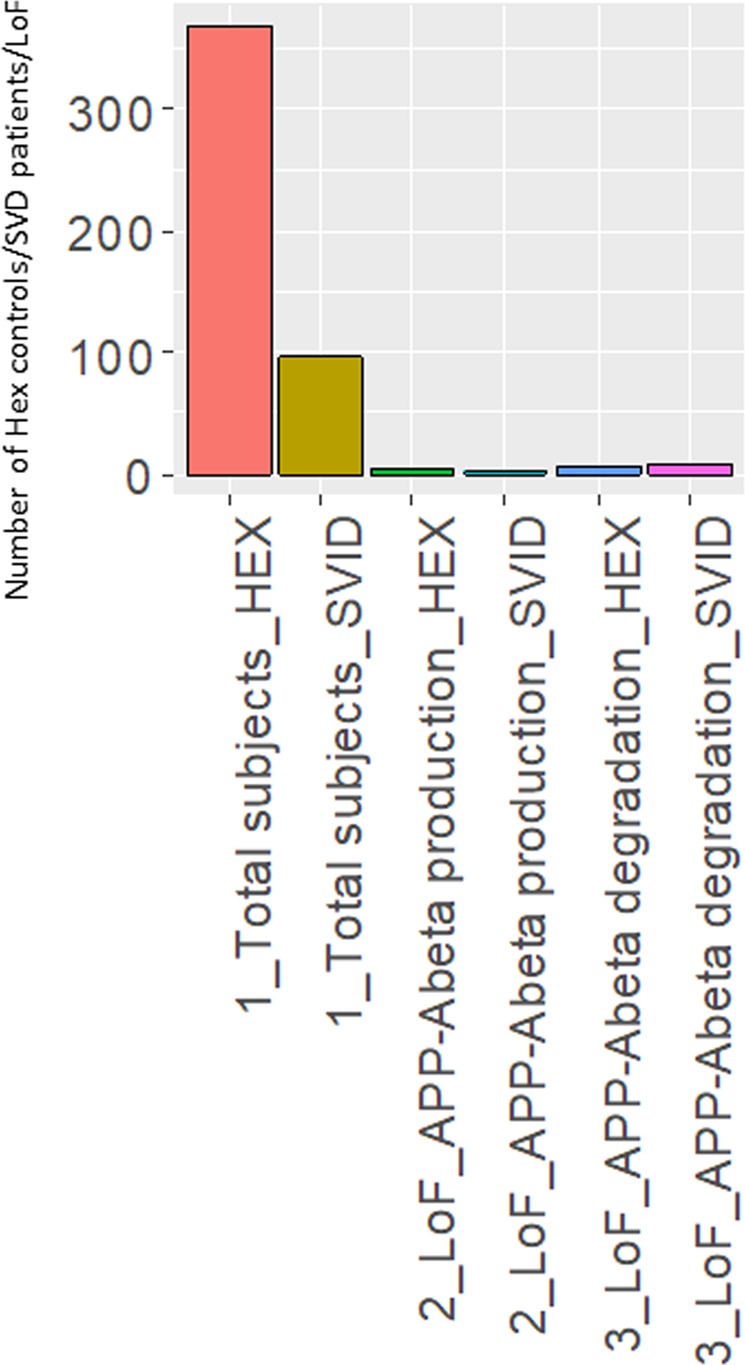
Number of loss of function (LoF) mutations in APP-Aβ degradation and production genes detected in the HEX and SVID cohorts and number of individuals per each cohort. The SVID cohort presents a burden of truncating mutations, compared to the HEX cohort. SVID, small vessel ischemic disease.

We report a significant enrichment for loss of function (LoF) mutations (stop gain/loss, inframe insertions/deletions, splice-site mutations) in genes regulating Aß degradation in SVID patients (9/96 [9.4%]), both when compared to APP-Aß production genes (1/96 [1%]) (Fisher p-value = 0.01837), and Aß degradation genes in the HEX cohort (6/368 [1.6%])(Fisher p-value= 3.496e-14) (Fig. 2, Table 4).

**Table 4:** Loss of function mutations detected in the SVID and HEX cohort.

Cohort	Gene	Position	rsID	Ref/Alt	Genomic change	Aa change	Carrier freq (%)	MAF(%)	Hex70 (%)
SVID	*MME*	3: 154834478	rs749320057	AC/A	c.467del	p.P156Lfs*14	1/96 (1)	0.5	0
SVID	*PLAT*	8: 42033519	rs1804182	G/A	c.1681C > T	p.R561*	1/96 (1)	0.5	—
SVID	*IDE*	10: 94238404	rs763710639	G/GGT	c.1881_1882insAC	p.L628Tfs*5	1/96 (1)	0.5	—
SVID	*IDE*	10: 94333763	rs533083105	AG/A	c.13del	p.L5fs	1/96 (1)	0.5	0.35
SVID	*MMP3*	11: 102713159	rs781898035	TACC/T	c.499_499 + 2del	p.E167del	1/96 (1)	0.5	—
SVID	*LRP1*	12: 57602503	NOVEL	C/A	c.12048 C > A	p.Y4016*	1/96 (1)	0.5	—
SVID	*LRP1*	12: 57603939	rs757410385	G/GC	c.12575dup	p.D4193Rfs*9	1/96 (1)	0.5	0.14
SVID	*MEP1B*	18: 29784212	rs200539508	C/T	c.436 C > T	p.R146*	1/96 (1)	0.5	0
SVID	*APP*	21: 27394181	rs764406483	TGTG/T	c.837_839del	p.T280del	2/96 (2)	1	—
SVID	*LRP1*	12:57605739	rs759104743	TTGC/T	c.13300_13302del	p.L4434del	1/96 (1)		
HEX	*ADAM17*	2:9630398		GATC/G		p.Asp794del	1/368 (0.3)	0.0014	0.0014
HEX	*BACE1*	11:117160453	rs758335005	G/T		p.Tyr445*	1/324 (0.3)	0.0015	0.0015
HEX	*APP*	21:27394181	rs768084853	TGTGGTGGTG/TGTGGTG		p.Thr280del	1/101 (1)	0.005	0.005
HEX	*APBA1*	9:72131320	rs768254638	GTCC/G		p.Glu268del	1/360 (0.2)	0.0014	0.0014
HEX	*ACE*	17:61562376		C/T		p.Gln50*	1/367 (0.2)	0.0014	0.0014
HEX	*CST3*	20:23615971		C/A		p.Glu93*	1/367 (0.2)	0.0014	0.0014
HEX	*CST3*	20:23615984	rs760409425	G/T		p.Tyr88*	1/366 (0.2)	0.0027	0.0027
HEX	*LRP1*	12:57605739	rs759104743	TTGC/T		p.Leu4432del	1/368 (0.2)	0.0014	0.0014
HEX	*MEP1B*	18:29796983		C/T		p.Gln597*	1/366 (0.2)	0.0014	0.0014
HEX	*ECE2*	3:184002778	rs769984677	G/T		p.Glu463Ter	1/368 (0.2)	0.0014	0.0014

Aa, amino-acid change. Freq, frequencies; MAF, minor allele frequency; SVID, small vessel ischemic disease.

### LOAD GWAS loci (*BIN1*, *CD33*, *MS4A6A*, *PICALM*, *CLU*, *CR1*, *EPHA1*, *ABCA7)*

*Single coding variant association test. EPHA1 p.V160A, CD33 p.A14V, ABCA7 p.G1527A are LOAD GWAS hits or in LD with LOAD GWAS hits and may play a modest protective effect in SVID.*

To investigate the role of the LOAD GWAS loci with the strongest effect sizes after *APOE* (*BIN1*, *CD33*, *MS4A6A*, *PICALM*, *CLU*, *CR1*, *EPHA1*, *ABCA7)*, we focus on a possible significant association between coding variants detected in these loci and SVID.

We screened protein coding variability in 9 highly replicated LOAD GWAS loci in 96 SVID patients and we identified 69 coding variants: 26 common, 4 rare and 39 very rare. Among these, 24 were singletons, 6 were novel and 6 were truncating mutations. *PICALM* and *CD2AP* did harbour the lowest number of variants (0,0005 variants per Kb of coding sequence). By contrast, *MS4A6A* harboured the highest number of coding variants (7,43 variants per Kb of coding sequence). The majority of patients, 81/96 (84.3%), carried at least one rare likely damaging variant and almost half of them, 45/96 (46.8%), harboured multiple likely pathogenic alleles in the studied genes.

Among these variants, 36/69 (52.2%) have been also detected in the 368 controls>70 years of age in the HEX database and were selected for the single-variant-based analysis (Table 4).

The single variant association test identified 3 coding variants whose allelic frequency significantly differed between SVID cases and controls (*EPHA1* p.M900V and p.V160A and *CD33* p.A14V) (adj. p-value <0.05). Importantly, all of these are common variants, with a modest to no damaging effect (15.46 > CADD score> 5.284) with moderate to strong likely protective effect size (0.3 < OR < 0.6) and only a minority of carriers are at least heterozygous for the *APOE* ε4 or ε2 alleles (up to 28.2% and 21.2%, respectively) (Table 5).

**Table 5:** Alzheimer’s disease GWAS hit single-variant association test in the SVID cohort.

Gene	Position	rs ID	Alleles	cDNA change	Aa change	MAF HEX_70	MAF SVD	CADD score	SVD_allele count/ allele number	HEX_allele count/ allele number	p-val	p-val adj	OR	CI	APOE ε4/ε2 (%)	Comment
*EPHA1*	7:143088867	rs6967117	T/C	c.2698 A > G	p.M900V	0.919	0.786	15.46	151/192	658/716	1.414e-06	5.0e-05	0.325	0.205–0.517	27.5/15	
*CD33*	19:51728477	rs12459419	C/T	c.41 C > T	p.A14V	0.3251	0.208	13.71	40/192	238/732	1.4e-03	0.025	0.546	0.363–0.808	27.2/21.2	LD with LOAD GWAS hit rs3865444 (Raj *et al*., 2014, p. 33) Blood Protein Level GWAS hit (Suhre *et al*., 2017) Hematological trait GWAS hit (Astle *et al*., 2016)
*EPHA1*	7:143097100	rs4725617	A/G	c.479 T > C	p.V160A	0.917	0.843	11.84	162/192	675/736	3.9e-03	0.04680	0.488	0.299–0.810	28.2/12.9	Blood Protein Level GWAS hit (MacArthur *et al*., 2017)
*ABCA7*	19:1056492	rs3752246	G/C	c.4580 G > C	p.G1527A	0.8388	0.755	5.284	145/192	609/726	1.06e-02	0.086	0.593	0.398–0.891	31.4/12.7	LOAD GWAS hit (Cuyvers *et al*., 2015)
*CR1*	1:207726161	rs200082366	G/T	c.3066 G > T	p.Q1022H	0.0936	0.036	15.51	7/192	38/406	0.012	0.086	0.366	0.135–0.852	16.6/50	protecting against immunocomplex deposition (Birmingham *et al*., 2003)
*CD33*	19:51728641	rs2455069	A/G	c.205 A > G	p.R69G	0.407	0.442	0.209	85/192	299/734	0.4108	1	1.155	0.826–1.611	32.3/15.38	Blood Protein Level GWAS hit (Suhre *et al*., 2017) Hematological trait GWAS hit (Astle *et al*., 2016)
*CR1*	1:207782916	rs4844609	A/T	c.4828 A > T	p.T1610S	0.974	0.994	6.589	191/192	717/736	0.095	0.570	5.056	0.793–211.22	29.1/13.5	
*CR1*	1:207795320	rs2296160	A/G	c.5905 A > G	p.T1969A	0.834	0.880	0.001	169/192	599/718	0.145	0.745	1.459	0.893–2.468	29.78/13.8	
*CR1*	1:207782931	rs6691117	A/G	c.4843 A > G	p.I1615V	0.1943	0.239	8.406	46/192	143/736	0.190	0.820	1.306	0.873–1.931	25/10	
*ABCA7*	19:1055191	rs3745842	G/A	c.4046 G > A	p.R1349Q	0.354	0.357	0.367	68/192	217/536	0.228	0.820	0.806	0.562–1.149	31.5/15.78	

ID, identification number; Aa, amino acid; MAF, minor allele frequency; SVID, small vessel ischemic disease; CADD, combined annotation dependent depletion; HEX, Healthy Exomes; p-val, p-value; adj, adjusted; OR, Odds Ratio; CI, Confidence Interval. LD, linkage disequilibrium, LOAD, late-onset alzheimer´s disease. GWAS, genome-wide association study.

Interestingly, *EPHA1* p.V160A, *CD33* p.A14V and 2 missense mutations nominally associated with SVID (*ABCA7* p.G1527A and *CR1* p.Q1022H) have been already reported either as functional, as LOAD GWAS hit, in linkage disequlibrium (LD) with LOAD GWAS hits or significantly linked to different complex traits (blood protein levels and haematological traits)^42,43^, further supporting the critical effect of the amino acid substitution in these positions. *EPHA1* p.V160A (rs4725617-G), was significantly associated with a moderate reduced risk for SVID (adj. p-value=0.046, OR = 0.488) and was located 12.04 kb proximal to rs11767557-T, significantly linked to LOAD and cognitive impairment^44^ and reported to be strongly associated to protein abundance levels^42,43^. In addition, *CD33* p.A14V, associated to *CD33* isoform lacking exon 2 and with a reduced inhibitory effect on microglia, has been reported to be in strong LD with the LOAD GWAS significant protective allele rs3865444-A^45,46^. *CR1* p.Q1022H has been shown to lower *CR1* expression and increase C4b binding, therefore protecting against immunocomplex deposition^47^. Moreover, *ABCA7* p.1527 G (rs3752246-C) is likely to reduce SVID susceptibility and, analogously, represents a LOAD GWAS hit and a modest protective allele for LOAD^48,49^.

## Gene expression screening during acute and subacute hypoperfusion in the BCCAS mouse model

### *APP-Aβmetabolism*

**Figure 3:**
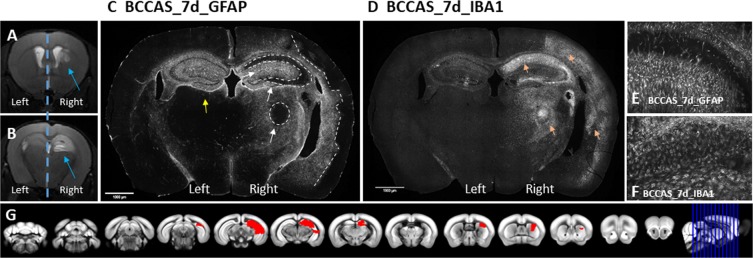
**A-G**.Bilateral common carotid artery stenosis (BCCAS) mouse model, displaying monolateral right small subcortical lesions, mainly affecting striatum (**A**) and hippocampus (**B**) (blue arrows), during acute (2d) and subacute (7d) hypoperfusion. The most severly hypoperfused hemisphere, with ischemic lesions detected on T2 weighted MRI (right hemisphere) was used for RNA sequencing. The contralateral hypoperfused hemisphere, with no ischemic lesions detectable on T2-MRI (left hemisphere) was used for immunohistochemistry. **C**, hypoperfused brain stained with GFAP, presenting peri-infarct astrocytosis in hippocampus and striatum during subcute hypoperfusion (7d) (white arrows). The infarct area is delimited by white dashed lines. The left hemisphere is hypoperfused and although ischemic lesions in the left side are not detectable on T2-MRI, we report a significant gliosis (yellow arrow). **D**, hypoperfused brain stained with IBA1, presenting microglia infiltration of the infarct areas and to a lesser extent peri-infarct areas and in the most severe hypoperfused areas during subcute hypoperfusion (7d) (pink arrows). **E**. Hypoperfused hippocampus stained with GFAP, displaying astrocytosis. **F**. Hypoperfused hippocampus stained with IBA1, displaying gliosis. **G**. T2-MRI slides showing the extension of the ischemic lesion (red).

We have used RNA sequencing data from prefrontal cortex, hippocampus and striatum of a mouse model of ischemia characterized by watershed and mainly subcortical infarcts (Fig. 3A–G), therefore a reliable model to study vascular dementia.

We detected a selective significant overexpression (up to 8-fold change and adj. p-value<0.05) of 5/17 genes (29.4%) involved in APP-Aß degradation (*A2m*, *Plat*, *Ctsd, Ctsb* and *Klk6*) and none of the genes controlling APP-Aß production during brain acute or subacute hypoperfusion (2d and 7d post-surgery, respectively). *A2m* was ubiquitously overexpressed both in prefrontal cortex, hippocampus and striatum, both 2 and 7 days post-surgery. By contrast, *Plat*, *Ctsd* and *Klk6*, displayed a specific time and region pattern of overexpression: *Plat* was overexpressed in prefrontal cortex and striatum 2 days post-ischemia and *Ctsd* and *Klk6* in striatum and hippocampus 7 days post-surgery, both tissues characterized by the highest degree of tissue remodelling and overall differential expression (Table 6).

**Table 6:** Differential gene expression during acute and subacute hypoperfusion in BCCAS mouse model.

Gene	AD path	Region_Time	Log2FC	P-value FDR
*A2m*	Aß catabolism	Hippocampus_2d	3.48	2.28E-14
		Prefrontal cortex_2d	3.07	2.10E-11
		Striatum_2d	2.57	3.56E-08
		Hippocampus_7d	2.9	3.98E-11
		Prefrontal cortex_7d	2.52	2.43E-07
		Striatum_7d	2.94	
*Plat*	Aß catabolism	Prefrontal cortex_2d	1.2	1.46E-09
		Striatum_2d	1.07	8.95E-08
*Klk6*	Aß catabolism	Hippocampus_7d	2.37	5.52E-08
		Striatum_7d		
*Ctsb*	Aß catabolism	Hippocampus_7d	1.4	2.50E-10
		Striatum_7d	1.3	8.37E-09
*Ctsd*	Aß catabolism	Hippocampus_7d	2.52	3.03E-14
		Striatum_7d	2.16	1.69E-10
*Apoe*	GWAS hit	Hippocampus_7d	1.35	4.20E-10
*Ms4a4a*	GWAS hit	Hippocampus_2d	1.8	2.93E-04
		Hippocampus_7d	1.85	6.08E-05
		Striatum_7d	1.61	6.80E-04
*Ms4a4c*	GWAS hit	Hippocampus_2d	2.56	3.65E-07
		Hippocampus_7d	2.08	1.48E-05
		Striatum_7d	2.27	1.89E-06
*Ms4a6c*	GWAS hit	Hippocampus_2d	1.76	2.86E-04
		Hippocampus_7d	2.8	4.64E-11
		Striatum_7d	2.32	8.45E-08
*Ms4a6d*	GWAS hit	Hippocampus_2d	2.53	1.43E-09
		Hippocampus_7d	2.33	3.98E-09
		Striatum_7d	1.98	9.37E-07
*Ms4a14*	GWAS hit	Hippocampus_7d	2.75	3.36E-09
		Striatum_7d	1.66	8.88E-04
*Ms4a4b*	GWAS hit	Hippocampus_7d	2.11	8.51E-06
		Striatum_7d	1.72	4.70E-04
*Ms4a6b*	GWAS hit	Hippocampus_7d	1.8	3.57E-07
		Striatum_7d	1.48	4.41E-05
*Ms4a7*	GWAS hit	Hippocampus_7d	2.65	1.50E-09
		Striatum_7d	1.89	3.89E-05
*CD33*	GWAS hit	Hippocampus_7d	2.18	1.52E-22
*Cd68*	Microglia marker	Hippocampus_2d	1.46	6.68E-04
		Hippocampus_7d	2.68	3.16E-13
		Striatum_7d	2.33	5.05E-10
*Aif1*	Microglia marker	Hippocampus_7d	2.25	5.64E-17
		Striatum_7d	1.57	1.16E-08

AD, Alzheimer’s disease; FDR, false discovery rate; GWAS, genome-wide association study.

### LOAD GWAS loci

**Figure 4:**
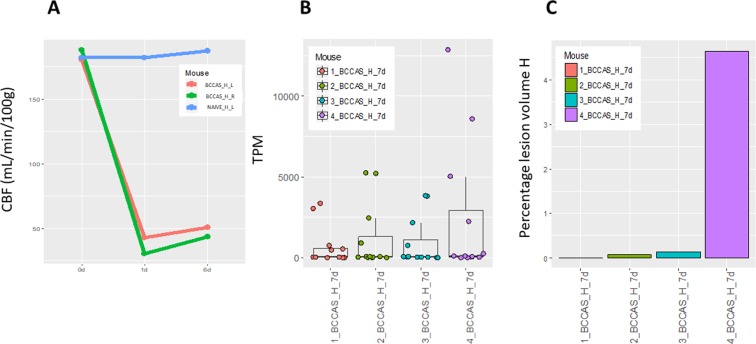
(**A**) Cerebral blood flow (CBF) reduction detected on MRI in hippocampus during acute (2d) and subacute (7d) hypoperfusion in BCCAS mice. The CBF drop at day 1 reaches 60–70% of the CBF values detected before the surgery (d0) or in naive mice and progressively recovers.CBF, cerebral blood flow; d, day; BCCAS, bilateral common carotid artery stenosis. (**B**) Differential gene expression, expressed in transcript per million (TPM) in APP-Aß metabolism genes and LOAD GWAS loci detected in BCCAS mice in hippocampus during subacute hypoperfusion (7d). **C** Percentage of ischemic lesion volume detected on T2-MRI in hippocampus of BCCAS mice during subacute hypoperfusion. H,hippocampus; L, left; R, right; d, day.

We report a significant upregulation (up to 7-fold change, and adj. p-value<0.05) of *Apoe*, *Cd33*, *Ms4a* cluster (*Ms4a4a, Ms4a4c, Ms4a6c, Ms4a6d, Ms4a14, Ms4a4b, Ms4a6b, Ms4a7*), particularly in the most affected brain areas during subacute hypoperfusion (hippocampus and striatum, d7) (Fig. 3). Together with APP-Aβ degradation genes (*A2m*, *Plat*, *Ctsd*, *Ctsb* and *Klk6*), the overexpression of *Apoe*, *Cd33*, *Ms4a* likely relied on microglia infiltration in the infarct and peri-infarct area at day 7 as these genes shared the same expression pattern of other microglia markers such as *Aif1* and *Cd86* (up to 6-fold upregulation in hippocampus and striatum), was proportional to the hippocampal lesion volume detected at day 7 on T2-weighted MRI and caused by a severe drop of brain cerebral blood flow (CBF) (≈60–70% brain CBF reduction compared to naive mice) (Fig. 4). Thus strongly arguing for consequential rather than causal upregulation of these genes and a possible role in ischemic lesion resolution. This was further supported by the concomitant significant co-expression of 7 different matrix metallo proteases (*Mmp2*, *Mmp8*, *Mmp11*, *Mmp12*, *Mmp13*, *Mmp19*, *Mmp25*) and 51 lysosomal genes at day 7 (Tables S5 and S6, Fig. S2C,D).

### Aβ oligomers detection during brain acute-subacute hypoperfusion in BCCAS mice

**Figure 5:**
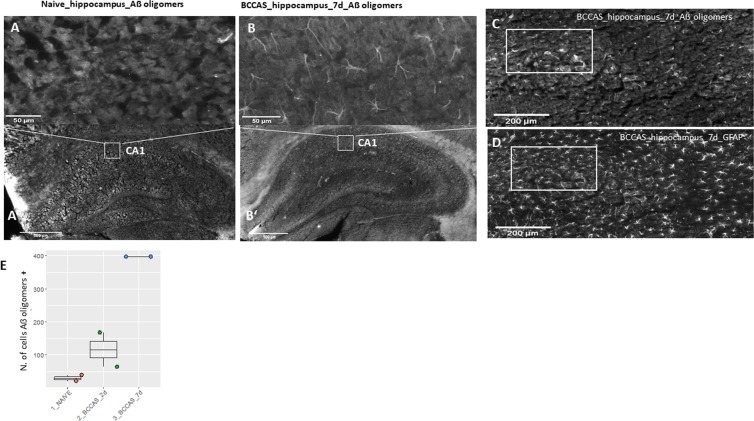
Toxic Aβ oligomers are detected in hypoperfused hippocampus mostly in the CA1 region during subacute hypoperfusion (7d) (**B,B’**) and absent in naive hippocampus (**A,A’**). The Aβ oligomers co-localize with reactive astrocytes (**C-D**). The number of glial and neuronal cells positive for Aβ oligomers are significantly higher in hippocampus during subacute hypoperfusion (7d) compared to acute hypoperfusion (2d) and naive mice (**E**). BCCAS, bilateral common carotid artery stenosis; d, day; +, positive.

We investigated the hypothesis that acute-subacute ischemia may have triggered the *de novo* misfolding of APP with a consequent generation of toxic Aβ oligomers. We indeed identified Aß oligomers mostly in CA1 region in the hippocampus of BCCAS mice 7 days post-surgery (Fig. 5A,B’). These were found both in pyramidal neurons (mainly axonal processes) (Fig. 5B-B’) and particularly in reactive astrocytes (Fig. 5C,D), analogously to those detected in APPPS1 mice at the age of 2 months (Fig. 3). These were present to a significant lower degree in the hippocampus of BCCAS mice 2 days post-surgery (t-test p-val=0.0321), characterized by a very moderate microglia/macrophage infiltration as suggested by *Cd68* and *Aif1* expression (Table 6), a modest overall tissue remodelling and gene differential expression (Fig S2) and almost absent in the hippocampus of naive mice (t-test p-val=0.0006) (Fig. 5E). Thus supporting the hypothesis that severe subacute brain hypoperfusion (60–70% CBF reduction), which however may not cause ischemic lesions detectable on T2-weighted MRI was necessary and sufficient to prime APP misfolding (Fig. 3A,B).

## Discussion

In this study we aimed at investigating the role of AD known pathogenic alleles and pathways: *APOE* ε4 allele, APP-Aβ metabolism genes and LOAD most replicated GWAS hits both in terms of genetic variability in a cohort of 96 familial and early-onset SVID patients and differential gene expression during acute and subacute hypoperfusion in the BCCAS mouse model resembling vascular dementia (Fig. 1).

In our cohort, around one third of the patients (29%) carried *APOE* ε4 allele. E3/ε4 and ε4/ε4 genotype frequency (27% and 1%, respectively) approximated the one reported in Caucasian controls (21.3% and 1.8%, respectively) and was significantly lower compared to the frequency reported in Caucasian LOAD patients (41.1% and 14.8%, respectively) (p-value = 0.05192 and 0.0003155 for ε3/ε4 and ε4/ε4 genotypes, respectively) (Table 2)^50^. Therefore suggesting, in concert with previous studies, that *APOE* ε4 allele may not critically influence the susceptibility to SVID^51,52^.

Moreover we report the prevalent role of APP-Aβ degradation genes upon genes involved in APP-Aβ production as well as Mendelian genes causative for Alzheimer’s disease (*APP*, *PSEN1* and *PSEN2*). We detected an enrichment for truncating mutations in genes playing a key role in APP-Aß catabolism, both when compared to genes controlling APP-Aß production in SVID patients or APP-Aß degradation genes in 368 neuropathologically confirmed elderly controls (9.4% and 1.6%, respectively) (Fisher p-value= 3.496e-14) (Fig. 2, Table 4). In addition, we report a common polymorphism in *CST3* (p.A25T), whose homozygous carrier frequency was significantly higher compared to HEX controls (4.16% and 1.63%, respectively). Interestingly, this polymorphism has been already associated to macular degeneration and LOAD^40^. The presence of one or two minor alleles increases LOAD risk and lowered the age at onset, in a fashion described both independent and dependent from *APOE* ε4 allele^39,53^. Although the SVID patients carrying in homozygosity p.*CST3* p.A25T displayed at the age at onset and diagnosis only a moderately lower MMSE score (MMSE 28 and 26), given the young age at onset (average 49.75years), we do not exclude that they may manifest a dementing phenotype later in life (average age at onset for the LOAD patients homozygous for *CST3* p.A25T was> 75 years^39,54^) (Table S3). The polymorphism is supposed to influence CST3 intracellular processing with a reduced extracellular secretion^55,56^, leading to increased amyloid fibril formation and Aß deposition^57^. A similar effect to what has been described for the pathogenic mutation *CST3* p.L68Q, causative for hereditary cerebral haemorrhage with amyloidosis, Icelandic type (HCHWA-I), resulting in increased intracellular localization of the mutant Cystatin C^58^.

Furthermore, we detected 2 rare coding variants (p.E270K and p.A528T) in the *SORL1* VPS10 domain, reported to interact with Aβ and harbouring *SORL1* pathogenic mutations^59^ (Table S1). The carrier frequency of *SORL1* variants in the VPS10 domain detected in our SVID cohort was similar to the frequency detected in a Caucasian British and American LOAD cohort, where 8 variants in 323 patients have been identified (2% and 2.4%, carrier frequency in SVID and LOAD, respectively) whereas only 5 *SORL1* variants in the VPS10 domain have been reported in 676 elderly controls in the same cohort (0.7%)^24^. This suggests that *SORL1* mutations may influence the susceptibility also for SVID and may support the previously reported role of *SORL1* in vascular dementia^60^.

By contrast, none of the 5 rare coding variants detected in *APP*, *PSEN1* and *PSEN2* are likely to be risk factors for SVID (Table 3). First, they have already been described as benign polymorphisms (*PSEN1* [p.E318G] and *PSEN2* [p.R62H]) (www.molgendatabase). Second, they all cluster outside the reported pathogenic domains (*APP* [p.V576I and p.T280del], *PSEN1* [p.E318G] and *PSEN2* [p.L2F and p.R62H]). This further shows that rare variants in *APP*, *PSEN1* and *PSEN2* are not common pathogenic factors in familial and early-onset apparently sporadic SVID cases. In line with this observation, 11/50 (22%) pathogenic mutations in *APP* have been reported as causative for AD and CAA, and only 2/50 (4%) lead exclusively to CAA. A smaller fraction of *PSEN1* pathogenic mutations (4/219 [1.8%]) has been described as causative for both AD and CAA and none exclusively for CAA. On the other hand, *PSEN2* harbours no causative mutation for CAA (www.molgendatabase).

The predominant role of APP-Aβ degradation genes was further confirmed by RNA sequencing data in a mouse model of mainly subcortical ischemia, mimicking small vessel ischemic disease, where only genes belonging to the APP-Aβ degradation path (*A2m*, *Plat*, *Ctsd*, *Ctsb* and *Klk6*) were significantly overexpressed in hippocampus and striatum during acute and subacute hypoperfusion (Table 6). Among these, *KLK6* expression has been already reported restricted to endothelial cells and increased of approximately 2-fold in the frontal cortex of patients with vascular dementia^61^.

Importantly, we showed that genetic and gene expression variability in LOAD GWAS genes are also likely to influence the susceptibility to SVID and acute-subacute ischemia.

We reported 3 common coding polymorphisms significantly associated to SVID and likely to play a mild protective role (adj. p-value <0.05 and 0.325<OR < 0.54): *EPHA1* p.M900V and p.V160A, *CD33* p.A14V (Table 5). Among these, *CD33* rs12459419-T has been reported to be in high LD with LOAD GWAS hit rs3865444-A and was suggested to explain its effect, such as the alternative splicing of CD33 with increased production of isoforms lacking exon 2, which encodes the IgV domain that typically mediates binding of sialic acid in SIGLEC family members. This CD33 isoform counteracts the inhibitory effect of CD33 on TREM2 in microglia and would ultimately reduce amyloid deposition and thus exert a moderate protective effect on Alzheimer’s disease and likely SVID susceptibility^46^. In addition, the critical role of this polymorphism (*CD33* rs12459419) was further reinforced by its significant association, together with *EPHA1* p.V160A, with blood protein levels, and haematological traits in two different GWASs^42,43^.

Moreover, *Apoe*, *Cd33* and the *Ms4a* cluster were significantly upregulated in hippocampus and striatum particularly during subacute hypoperfusion (up to 7-fold change at day 7)(Table 6). Together with APP-Aβ degradation genes, overexpression of *Apoe*, *Cd33* and *Ms4a* cluster correlated with hippocampal lesion size at day 7 (Fig. 4B,C) and likely microglia infiltration of the infarct and peri-infarct areas (co-expression of microglia markers *Cd68* and *Aif1*), suggesting that upregulation of these genes was tightly driven by and consequential to the severity of hypoperfusion, moreover, arguing for an active role of these genes in tissue remodelling and ischemic lesion resolution. This is further supported by the fact that significant overexpression of *Cd33*, *Ms4a6d* and *Apoe* (> 1.5 fold change and adj p-value <0.05) markedly correlated with AD pathology in 2 mouse models of Alzheimer’s disease characterized by severe Aβ plaques and tau tangle deposition (HOTASTPM and TAU mice, 18 months of age)^62^. Therefore implying that *Cd33*, *Ms4a* cluster and *Apoe* are not Aβ or tau specific.

Finally, the shared pathogenic pathway between LOAD-SVID-ischemic stroke was supported by histological findings of neurons and reactive astrocytes positive for Aβ oligomers in the main hypoperfused areas such as hippocampus at day 7 in the BCCAS mouse model (Fig. 5).

Previous studies in stroke experimental models (middle cerebral artery occlusion [MCAO]) reported Aβ in reactive astrocytes and neurons and Aβ plaque-like deposits in peri-infarct areas: particularly corpus callosum, CA1 hippocampal areas and mainly subcortical areas 7 days post-surgery^63,64^. The authors hypothesized that Aβ may have been the result of APP overexpression during ischemic stress^65^. Importantly, we show that acutely-subacutely hypoerfused brain areas and particularly reactive astrocytes and hippocampal neurons are positive for Aβ oligomers rather than β amyloid, that it is likely to represent a late and chronic event in the APP misfolding cascade. Moreover, we detected Aβ oligomers in the hypoperfused brain regions, displaying gliosis but not necessarily gray or white matter hyperintensities detectable on T2-weighted MRI (Fig. 5, Fig. 3A,B). Thus suggesting that a marked degree of hypoperfusion-ischemia that may remain however below the T2-weighted MRI detectability and does not lead to strokes, may trigger APP misfolding and may explain the link between brain microstructural changes detected on diffusion tensor imaging (DTI) and likely hypoxic-ischemic hyperintensities in white matter, detected decades before the onset of symptomps and autosomal dominant AD cases^66,67^ as well as common late-onset sporadic cases and elderly people^68^.

Therefore, our data may unveil at least some of the pathogenic mechanisms by which ischemic stroke may precipitate the progression of AD in experimental models and patients and why cerebrovascular accidents may accelerate AD onset particularly in asymptomatic elderly patients with AD pathology^69^. Indeed, the enrichment for genetic variability in APP-Aβ degradation genes has been reported playing a key role in sporadic late-onset AD whereas increases in Aβ production currently explain a minority of AD cases^37,70^.

In summary, we provide genetic, gene-expression and histological data supporting a shared pathogenic ground between LOAD and SVID-acute ischemia. Our genetic data in SVID patients, together with expression data in a vascular dementia mouse model show that 1) *APOE* transcriptional regulation but not ε4 allele may play a role in brain hypoperfusion and small vessel ischemic disease; 2) APP-Aβ degradation plays a prevalent role upon APP-Aβ production; 3) *APP*, *PSEN1* and *PSEN2* are not common pathogenic factors in SVID; 4) *CD33*, *CR1*, *EPHA1* and the *MS4A* cluster may be involved in SVID and brain subacute hypoperfusion-ischemia and 4) acute and mainly subacute ischemia may trigger Aβ toxic oligomer formation. Thus suggesting that the vascular hypothesis^71^ and the amyloid cascade hypothesis^72^ in AD may complement each other, rather than being mutually exclusive. Our findings warrant further genetic screening in a larger cohort and functional studies.

## Supplementary information

Supplementary Figure S1

Supplementary Figure S2

Supplementary Figure S3

Supplementary Information

Supplementary Tables

## Data Availability

All data generated or analysed during this study are included in this published article (and its Supplementary Information files).
