# Supplementary figures and images for: Investigating APOE, APP-Aβ metabolism genes and Alzheimer’s disease GWAS hits in brain small vessel ischemic disease
Source: Sci Rep. 2020 Apr 28;10:7103. doi: 10.1038/s41598-020-63183-5 (PMC7188838; doi:10.1038/s41598-020-63183-5)

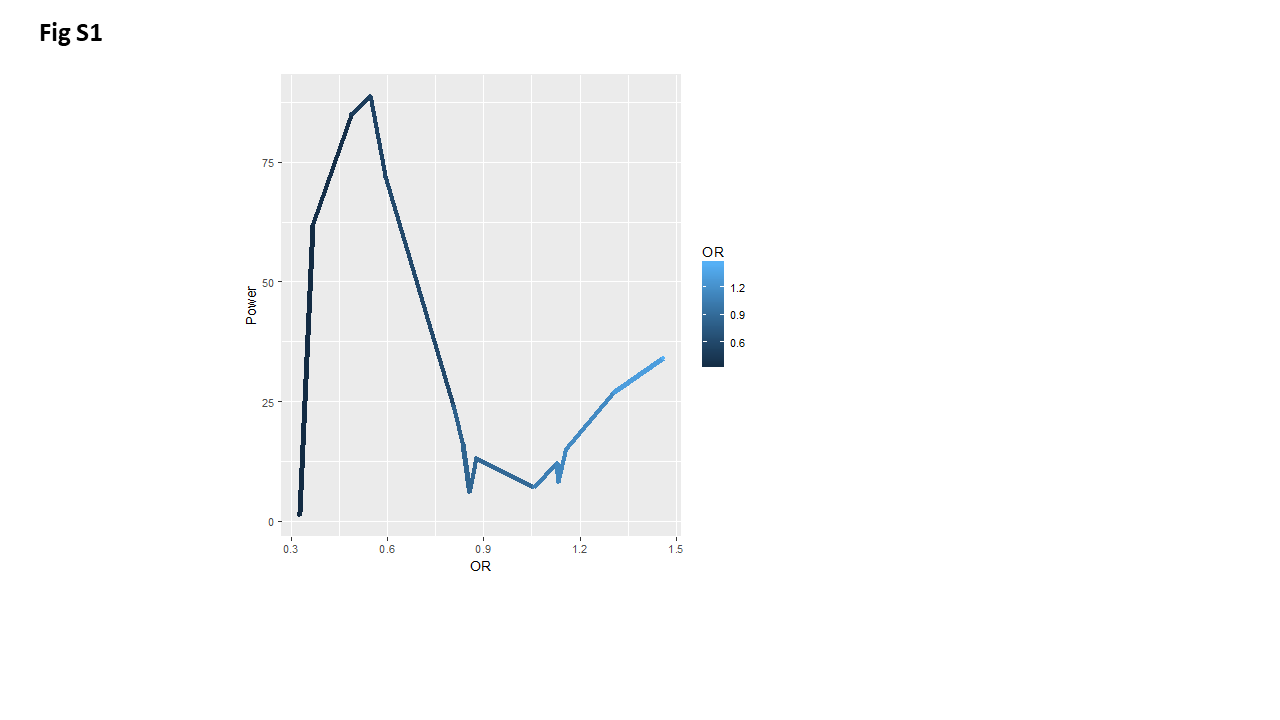

Supplement: Supplementary file 1 — Supplementary Figure S1 [file 41598_2020_63183_MOESM1_ESM.tif]

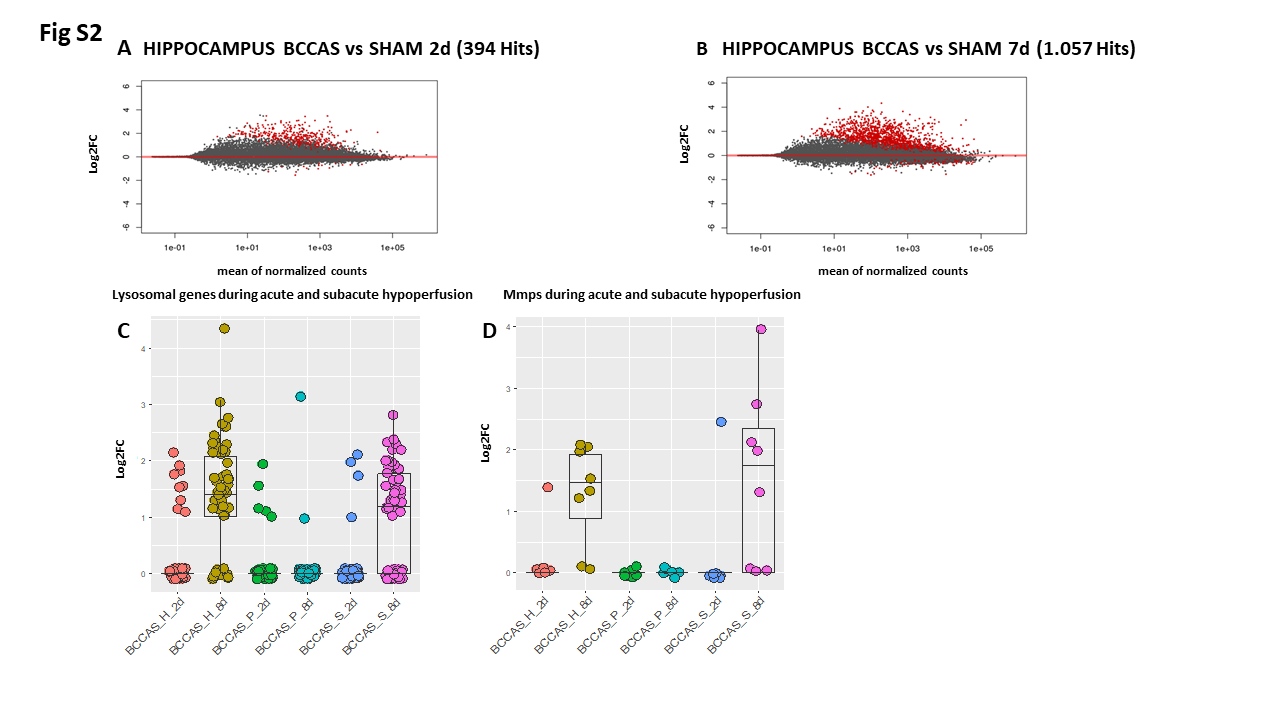

Supplement: Supplementary file 2 — Supplementary Figure S2 [file 41598_2020_63183_MOESM2_ESM.tif]

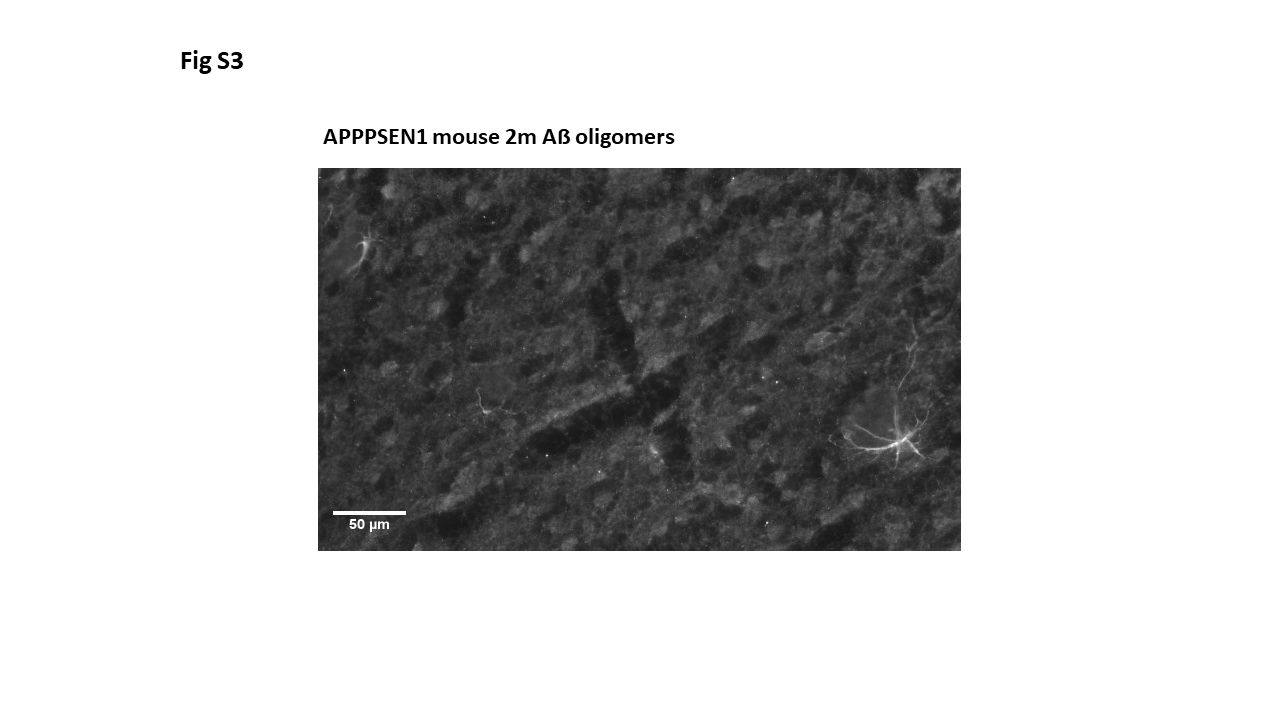

Supplement: Supplementary file 3 — Supplementary Figure S3 [file 41598_2020_63183_MOESM3_ESM.tif]
