# Supplementary material for: Investigating APOE, APP-Aβ metabolism genes and Alzheimer’s disease GWAS hits in brain small vessel ischemic disease
Source: Sci Rep. 2020 Apr 28;10:7103. doi: 10.1038/s41598-020-63183-5 (PMC7188838; doi:10.1038/s41598-020-63183-5)
Supplement: Supplementary file 4 — Supplementary Information [file 41598_2020_63183_MOESM4_ESM.docx]

Investigating *APOE*, APP-Aβ metabolism genes and Alzheimer’s disease GWAS hits in brain small vessel ischemic disease

Sonja Blumenau^1**^, Marco Foddis^1**^, Susanne Müller^1^, Manuel Holtgrewe^2^, Bentele Kajetan^2^, Daniel Berchtold^1^, Dieter Beule^2^, Ulrich Dirnagl^1^, Celeste Sassi^1*^

^1^Department of Experimental Neurology, Center for Stroke Research Berlin (CSB), Charité - Universitätsmedizin Berlin, Corporate Member of Freie Universität Berlin, Humboldt-Universität zu Berlin, and Berlin Institute of Health, Berlin, Germany

^2^Berlin Institute of Health, BIH, Core Unit Bioinformatics, Berlin, Germany.

**= equal contribution

*=Corresponding author:

Celeste Sassi, MD, PhD

Klinik und Poliklinik für Neurologie
Abteilung für Experimentelle Neurologie
Charité – Universitätsmedizin Berlin

Charitéplatz 1

D-10117 Berlin

Tel.: 030 – 450 560 149

Fax: 030 – 450 560 915

[celeste.sassi@charite.de](mailto:celeste.sassi@charite.de)

**SUPPLEMENTARY MATERIALS AND METHODS**

**BCCAS**

As previously described^1^, anaesthesia was achieved using isoflurane in a 70:30 nitrous oxide:oxygen mixture and core body temperature was maintained at 37 ± 0.2 °C with an automated rectal probe and heat blanket. A midline incision was made in the neck, and a carotid artery was carefully exposed. Hypoperfusion was induced by winding a custom ordered, non-magnetic, surgical grade microcoil (160 µm inner diameter, Shannon Coiled Springs Microcoil, Limerick, Ireland) around one of the carotid arteries. The sham procedure was performed with a larger diameter microcoil (500 µm) that did not constrict the vessel. The muscle and glands were guided back into place and local anaesthetic was applied to the sutured wound prior to recovery. Twenty- four hours later, the same procedure was repeated on the other carotid artery. This delay represents an important refinement that does not result in higher mortality when using the smaller sized microcoils. Regular diet was placed on the floor of the cage to assist with feeding, and animals were provided with 6 mg/mL of Paracetamol in the drinking water to assist with post-operative pain (one day prior to, and up to three days post-surgery).In the BCCAS model, hypoperfusion was induced by winding a custom ordered, nonmagnetic, surgical grade microcoil (160 μm inner diameter; Shannon Coiled Springs Microcoil, Limerick, Ireland) around one of the common carotid arteries. The muscles and glands were guided back into place, and local anesthetic was applied to the sutured wound before recovery. Twenty-four hours later, the same procedure was repeated on the other common carotid artery. This delay represents an important refinement that does not result in higher mortality when using the smaller sized microcoils.

**MRI measurements**

Anaesthesia was again achieved using isoflurane as per above, and body temperature and respiration rate were monitored with MRI compatible equipment (Small Animal Instruments, Inc., Stony Brook, NY).

*Cerebral blood flow and angiographies*

CBF and angiography were measured on a 7 T Pharmascan using Paravision 5.1 software (Bruker BioSpin, Ettlingen, Germany). For the CBF measurement, radio frequency transmission was achieved with a 72 mm diameter quadrature resonator actively decoupled to a mouse quadrature surface coil used for reception (Bruker BioSpin, Ettlingen, Germany). A single slice (1 mm) flow-sensitive alternating inversion recovery (FAIR) sequence with a rapid acquisition with relaxation enhancement (RARE) readout was used (repetition time (TR)/recovery time/echo spacing (ΔTE)/effective echo time (TEeff): 12 000/10 000/7.2/35.9 ms, respectively, 16 inversion times (35-1500 ms), RARE factor: 32, inversion slice thickness: 4 mm, 180° hyperbolic secant (sech80) inversion pulse (20 ms), field of view (FOV): 25.6 mm2, matrix: 128 x 64 enlarged by partial fourier transform to 128 x 128, resolution: 200 µm2, 12 min). For angiography measurements, a 20 mm diameter quadrature volume coil (RAPID Biomedical, Rimpar, Germany) was used for radio frequency transmission and reception and a 3D time of light (TOF) sequence was used (TR/TE: 15/2.5 ms, α: 20 ˚, FOV: 25 mm3, resolution: 98 x 130 x 196 µm3 zero-filled to 98 µm3, 6 min). Spectroscopy, T2 weighted and MR spectra were acquired on a 7 T Biospec with a cryogenically cooled transmit/receive surface coil and Paravision 6.0 software (Bruker BioSpin, Ettlingen, Germany).

A 2D RARE T2 sequence was used for anatomical images (TR/ΔTE/TEeff: 3100/11/33 ms, RARE factor: 8, 29 consecutive slices, slice thickness 0.45 mm, FOV: (16.2 mm)2, resolution: 100 µm2, NA: 2, 2 min 4 s). A stimulated echo acquisition mode (STEAM) sequence was used for spectroscopy following local shimming (MAPSHIM) across a cubic 8 mm3 voxel placed in the striatum (TR/TE/mixing time: 2500 ms/3 ms/10 ms, number of averages (NA): 256, VAPOR water suppression, 10 min 40 s).

**MRI Data Analysis**

CBF maps were calculated using the Perfusion ASL macro in Paravision 5.1 software via the T1 method using a blood T1 value of 2100 ms and a brain blood partition coefficient of 0.89 mL/g ^2^, ^3^. Analysis of the CBF values were done using a custom written Matlab toolbox for nonlinear atlas registration ^4^ was used to select the CBF slice from the volume and coregister the CBF on the T2 images. Finally T2 and CBF images were transformed into the Allen brain atlas space and the atlas based CBF-values were extracted for all correlating Allen brain atlas structures in both hemispheres. (Release 2013a (MathWorks, Natick, MA, USA) script extracted the CBF maps from Paravision, and used atlas registration and coregistration of CBF maps in the atlas space for striatum and prefrontal cortex). The resulting CBF values were expressed in mL/min/100g.

**References**

1. Foddis, M. *et al.* An exploratory investigation of brain collateral circulation plasticity after cerebral ischemia in two experimental C57BL/6 mouse models. *J Cereb Blood Flow Metab* 0271678X19827251 (2019) doi:10.1177/0271678X19827251.

2. Leithner, C. *et al.* Determination of the brain-blood partition coefficient for water in mice using MRI. *J. Cereb. Blood Flow Metab.* **30**, 1821–1824 (2010).

3. Dobre, M. C., Uğurbil, K. & Marjanska, M. Determination of blood longitudinal relaxation time (T1) at high magnetic field strengths. *Magn Reson Imaging* **25**, 733–735 (2007).

4. Koch, S. *et al.* Atlas registration for edema-corrected MRI lesion volume in mouse stroke models. *J. Cereb. Blood Flow Metab.* 271678X17726635 (2017) doi:10.1177/0271678X17726635.

**Figure S1**

Power calculation in our SVID cohort, showing that the study had 80% power to detect common variants with strong effect size. R statmod-package [v1.4.32](https://www.rdocumentation.org/packages/statmod/versions/1.4.32) for Fisher´s exact test based on allelic association was used for this power calculation. OR, odds ratio.

**Figure S2**

**A-B**Differential gene expression detected during acute (2d)(**A**) and subacute (7d) (**B**) hypoperfusion in hippocampus of BCCAS mice. **C-D** Differential gene expression in lysosomal genes and matrix metalloproteases (Mmps) genes detected during acute and subacute hypoperfusion in the hippocampis of BCCAS mice.

**Figure S3**

Aβ oligomers detected in APPPS1 mice, before the appearance of amyloid plaques. These were analogous to the ones detected in BCCAS mice in hypoperfused hippocampi (7d)
